# Limitations of Stem Cell Therapy for Parkinson’s Disease

**DOI:** 10.3390/cells15090839

**Published:** 2026-05-03

**Authors:** Zhuowei Li, Shijun Peng, Jia Ouyang, Ruen Liu

**Affiliations:** 1 Peking University First School of Clinical Medicine, Peking University Health Science Center, Beijing 100191, China; 2110305122@stu.pku.edu.cn; 2 Department of Neurosurgery, Peking University People’s Hospital, Beijing 100044, China; genepeng@126.com (S.P.); ouyangjiashen@126.com (J.O.)

**Keywords:** Parkinson’s disease, stem cell therapy, gene therapy

## Abstract

**Highlights:**

**What are the main findings?**

- Stem cell therapy for Parkinson’s disease faces multiple limitations as follows: it fails to ameliorate non-motor symptoms unrelated to dopaminergic dysfunction, autologous cells may retain disease-associated genetic defects, transplanted cells remain vulnerable to the host’s pathological microenvironment, and ectopic transplantation prevents the establishment of appropriate afferent neural inputs.
- The therapeutic efficacy of stem cell transplantation is profoundly influenced by patient-specific factors—including age, disease stage, duration, and levodopa responsiveness—and the integrity of the host’s nigrostriatal system, defining a limited patient population that may derive optimal benefit.

**What are the implications of the main findings?**

- Orthotopic transplantation and combining stem cell therapy with complementary strategies—such as gene editing to confer resistance to α-synuclein pathology, neuroprotective interventions to modulate the host microenvironment, and adjunctive physical training—may overcome the current limitations and enhance graft survival, integration, and functional durability.
- Precise patient stratification based on disease parameters and emerging biomarkers, coupled with further mechanistic research into PD pathogenesis and neural circuitry, will be essential to translate stem cell therapy from a symptomatic treatment into a more comprehensive and personalized therapeutic approach.

**Abstract:**

Stem cell therapy, as a potential treatment for Parkinson’s disease (PD), has been investigated in many clinical trials for its effectiveness. However, the therapeutic outcomes have shown considerable variability. This may be due to certain limitations of stem cell therapies, such as the inability to ameliorate non-motor symptoms or modify underlying disease mechanisms, and the limited applicability in certain patient populations. Combinations with the recently emerging gene therapy and other available technologies are expected to make up for or break down these limitations of stem cell therapy, thereby broadening its scope of application. In this review, we will discuss the limitations of stem cell therapy in terms of pathogenesis, symptoms, and population, as well as possible coping strategies to enhance the effectiveness of stem cell therapy.

## 1. Introduction

Parkinson’s disease (PD) is the second most prevalent neurodegenerative disorder after Alzheimer’s disease, affecting approximately 12 million people worldwide [1,2], and it is characterized by the progressive degeneration of mesencephalic dopaminergic (mDA) neurons in the substantia nigra pars compacta (SNc) [3,4]. Striatal dopamine (DA) deficiency resulting from neuronal loss leads to resting tremor, rigidity, and bradykinesia, thereby impairing movement amplitude and speed [5]. Recent evidence suggests that neurons begin to lose their normal function and morphology even before they die, suggesting that simply stopping these neurons from dying is unlikely to be an effective treatment [4]. This means that, unlike many neurodegenerative diseases, the supplementation of dopaminergic neurons that are gradually lost in local brain regions is considered one of the most promising strategies to alleviate motor symptoms in patients with PD [6]. As a result, PD is emerging as a clinically promising target disease for stem cell therapy.

Stem cell therapy, a key approach within regenerative medicine, is believed to replace the dysfunctional dopaminergic innervation with dopamine-producing cells in hopes to restore lost neuronal circuitry caused by the focal degeneration of mesencephalic dopaminergic neurons [7]. It is considered to have potential value in related disorders with limited therapeutic options, such as atypical parkinsonian syndromes [8]. A large body of accumulated data has demonstrated the potential of cell transplantation to offer significant and long-term recovery from PD pathology [9]. Early clinical trials using human fetal ventral mesencephalic (fVM) tissue, initiated in Lund in the late 1980s, provided encouraging proof of concept for cell transplantation in PD. Although clinical outcomes were variable, a subset of patients exhibited marked and sustained motor improvement, including normalization of striatal DA levels and, in exceptional cases, complete withdrawal of anti-parkinsonian medication for more than two decades following transplantation [10]. More recently, building on earlier fVM tissue grafting experience, a first-in-human phase I/IIa trial using Good Manufacturing Practice (GMP)-manufactured human fetal midbrain-derived dopaminergic neural precursor cells demonstrated acceptable safety and dose-dependent motor improvement in patients with idiopathic PD over 12 months of follow-up [11]. In 2006, Takahashi and Yamanaka successfully reprogrammed human fibroblasts into a pluripotent cell line using the following four transcription factors: c-Myc, Oct3/4, Klf4, and Sox2. The induced pluripotent stem cell (iPSC) technology offers the unprecedented possibility of generating patient-specific transplantable dopaminergic progenitor cells without destroying the early embryo [12]. Using specific differentiation strategies, human iPSC-derived dopaminergic neurons can efficiently engraft, integrate into host striatal circuits, and functionally restore motor deficits in animal models of PD [13,14]. Recent preclinical progress has further advanced the field, exemplified by the development of the GMP-manufactured human embryonic stem cell (hESC)-derived dopaminergic progenitor product STEM-PD, which demonstrates robust quality, safety, and efficacy profiles, including long-term tumor-free survival, appropriate target innervation, and full motor recovery in rat PD models. A first-in-human phase I/IIa clinical trial using an in vivo-tested batch of STEM-PD has now been initiated, marking an important step toward standardized and scalable stem cell-based therapies for PD [15,16].

However, not all clinical trials of therapy based on dopaminergic neuron replacement have demonstrated consistent effectiveness. A recent phase I, open-label clinical trial investigated the safety, tolerability, graft survival, and potential efficacy of bemdaneprocel (a cryopreserved hES cell-derived dopaminergic neuron progenitor product) bilaterally grafted into the putamen of PD patients. Despite several post-transplant indicators showing some improvement, the study still presented signs of limited efficacy, including no significant improvement or even slight worsening of some motor and quality-of-life scores in the low-dose group, inconsistent changes in non-motor symptoms, no reduction in medication use or harmful movements (dyskinesia), and a large degree of individual variability in overall improvement across the small sample size [17].

These observations raise important questions regarding the specific conditions under which stem cell therapies for PD are therapeutically effective and the circumstances in which their limitations become apparent. Representative clinical trials and their key outcomes, durability, and limitations are summarized in Table 1. In this review, we address the existing clinical trials of stem cell therapy for PD, focusing on the limitations of the therapy in terms of efficacy after successful stem cell transplantation, thus providing ideas for the improvement of stem cell therapy or the creation of synergistic therapies in combination with it. In addition, we propose solutions and therapeutic modalities that can be realized based on the present technologies.

**Table 1:** Summary of representative clinical trials of stem cell therapy for PD.

Study (Ref.)	Publication Year	Cell Type	Trial Phase	Sample Size ^1^	Follow-Up Duration	Motor Outcome and Interpatient Variability	Non-Motor Outcome	Durability of Effect	Key Limitations
Lund fVM ^2^ trials [18,19,20,21]	1989–1994	fVM tissue	-	18	4–24 years	Modest early benefit in the first 2 patients at 6 months, but marked and sustained motor improvement in some patients during subsequent follow-up, including reduced OFF ^3^ time and, in one patient, L-dopa withdrawal after 32 months	Limited/uncertain non-motor benefit; possible cognitive decline in long-term follow-up	Sustained long-term symptomatic benefit in some patients; possible gradual decline in very long-term follow-up	Graft-induced dyskinesia; variable outcomes; limited effect on non-motor symptoms; host pathology may progressively affect graft efficacy
Freed et al. [22]	2001	Embryonic midbrain-derived dopaminergic neurons	-	20 grafted; 20 sham	12 months	No significant overall benefit on the primary global rating outcome at 12 months; improvement in off-medication UPDRS ^4^ in transplanted younger patients (≤60 years), but not in older patients	No significant difference in diary scores at 12 months	Limited overall benefit at 12 months; greater improvement with continued follow-up up to 3 years, especially in younger patients	Graft-induced dyskinesia/dystonia; benefit depended on age/patient selection
Olanow et al. [23]	2003	Fetal nigral tissue	-	23 grafted; 11 sham	24 months	No significant overall treatment effect in UPDRS motor OFF score; placebo worsened by 9.4, one-donor group by 3.5, and four-donor group improved by 0.72 points; benefit mainly in milder patients, especially in the four-donor group	No significant difference in most secondary endpoints (including activities of daily living, patient diaries, and quality-of-life measures)	Limited and non-uniform improvement; most PET ^5^ improvement by 12 months, with minimal additional increase at 24 months	Graft-induced dyskinesia; benefit limited mainly to selected milder patients; no clear overall efficacy
Kim et al. [11]	2023	Fetal midbrain- derived dopaminergic precursor cells	Phase I/IIa	15	12 months	Significant improvement in UPDRS Part III in the medium- and high-dose groups (up to 26.16% and 40%, respectively); no significant UPDRS Part III benefit in the low-dose group	No remarkable changes in emotional or neurocognitive function; no significant improvement in quality of life	Short-term benefit at 12 months; long-term durability not yet determined	Limited effect on non-motor symptoms; therapeutic benefit may depend on dose and patient selection
Tabar et al. [17]	2025	hESC ^6^-derived dopaminergic progenitors	Phase I	12	18 months	MDS-UPDRS ^7^ Part III OFF improvement of 8.6 points in the low-dose cohort and 23.0 points in the high-dose cohort at 18 months; greater amplitude of improvement and earlier onset in the high-dose group	No clear overall NMSS ^8^ improvement; PDQ-39 ^9^ improvement in the high-dose cohort and slight worsening in the low-dose cohort	Short-term benefit through 18 months; longer-term durability uncertain, with ongoing follow-up planned for at least 5 years	Limited effect on non-motor symptoms; therapeutic benefit may depend on dose and patient selection
Kyoto trial [24]	2025	iPSC ^10^-derived dopaminergic cells	Phase I/II	7 for safety; 6 for efficacy	24 months	Improvement in 4 of 6 patients on MDS-UPDRS Part III OFF; mean change −9.5 points (−20.4%) at 24 months, with score changes ranging from −50.0% to 8.5%; improvement in 5 of 6 patients on MDS-UPDRS Part III ON ^11^	No improvement in non-motor symptoms as assessed by MDS-UPDRS part I; on average, no apparent improvement in PDQ-39 at 24 months	Short-term benefit through 24 months; longer-term durability uncertain	Limited effect on non-motor symptoms; variable clinical efficacy despite positive 18F-DOPA uptake; therapeutic benefit may depend on age, disease severity, and progression of non-dopaminergic pathology
TransEuro trial [25]	2025	fVM tissue	-	11 grafted; 16 controls	36 months	No overall clinical benefit for the primary endpoint (UPDRS Part III OFF at 36 months); modest benefit in only some patients, with outcomes varying by surgical site/device and possibly disease stage	No clear broad non-motor benefit; only modest PDQ-39 change	Modest dopaminergic PET improvement at 18 months; near-normal restoration in only one patient	Mild graft-induced dyskinesia still occurred; outcomes remained variable because of patient selection, surgical/device factors, and poor standardization/tissue supply

^1^ Sample size is presented as total n for single-arm studies and as group-wise n for controlled studies. ^2^ fVM, fetal ventral mesencephalic; ^3^ OFF, off-medication state; ^4^ UPDRS, Unified Parkinson’s Disease Rating Scale; ^5^ PET, positron emission tomography; ^6^ hESC, human embryonic stem cell; ^7^ MDS-UPDRS, Movement Disorder Society-sponsored revision of the Unified Parkinson’s Disease Rating Scale; ^8^ NMSS, Non-Motor Symptoms Scale; ^9^ PDQ-39, Parkinson’s Disease Questionnaire-39; ^10^ iPSC, induced pluripotent stem cell; ^11^ ON, on-medication state; L-dopa, levodopa.

## 2. Limitations

In existing clinical trials of stem cell therapies for PD, even after successful stem cell transplantation, some Parkinson’s symptoms remain refractory, and stem cell therapies may fail to provide therapeutic benefit under certain conditions, leading to limitations in the application of stem cell therapies. This section summarizes these limitations, illustrated by evidence from clinical trials. Reviewing the limitations of this therapy will allow us to improve on the shortcomings of this therapy, as well as to combine new therapies that complement it, to achieve a comprehensive and effective treatment.

### 2.1. Non-Motor Symptoms

PD is a multisystem disorder. The pathology in PD involves multiple cell types not only within the central nervous system (CNS) but also including the autonomic and enteric nervous systems [26,27]. In CNS, besides the loss of dopaminergic neurons, serotonergic, noradrenergic, and cholinergic neurons are also affected [28]. These widespread pathological changes contribute to a wide range of non-motor symptoms, such as autonomic dysfunction (e.g., constipation, bladder dysfunction, orthostatic hypotension, and erectile dysfunction), neuropsychiatric symptoms (e.g., depression, cognitive dysfunction, and dementia), and sleep disorders (e.g., insomnia, rapid-eye movement sleep behavior disorder, hypersomnia) [29,30,31]. Cognitive impairment and dementia have been reported in 80% of PD patients 15–20 years after disease onset [32]. These changes may appear in the early stages of the disease, known as the premotor phase of Parkinson pathology, and may precede the onset of substantial irreversible cell loss by decades and then worsen as the disease progresses [33].

While there is a relationship between motor symptoms and Lewy bodies (LBs) and α-synuclein (α-syn) deposition, many non-motor changes do not correlate with the burden of α-syn inclusions in dopaminergic neurons, especially in cognitive and mental functions, suggesting that there may be different mechanisms involved in the degenerative process of non-motor function [34]. For example, cognitive impairment in PD has been associated with structural and functional alterations extending beyond the nigrostriatal system, with early involvement of cortical and subcortical regions such as the prefrontal cortex, temporal lobe, amygdala, thalamus, and hypothalamus [35,36]. These changes potentially involve multiple mechanisms beyond α-syn pathology, including widespread neural circuit dysfunction, disruption of neurotransmitter systems, failure of compensatory processes, and the presence of coexisting pathologies [35]. Among them, coexisting pathologies, including regionally selective β-amyloid deposition and tau pathology, may further aggravate cognitive dysfunction by increasing network vulnerability, impairing synaptic integrity, and destabilizing large-scale neural networks [37,38]. Moreover, mitochondrial dysfunction has emerged as a key contributor to synaptic failure and neuronal vulnerability in brain regions mediating cognition, promoting cognitive decline through impaired energy metabolism and disrupted synaptic transmission [39]. Some autonomic and homeostatic non-motor symptoms, such as thermoregulatory dysfunction, have been shown to be related to α-syn pathology; however, unlike classical nigrostriatal involvement, these alterations are characterized by α-syn deposition in hypothalamic and brainstem nuclei, together with altered functional connectivity within thermoregulatory networks and disrupted neurotransmitter modulation [26]. The coexistence of these diverse factors makes it difficult to associate non-motor symptoms with pathological changes in individual neuronal populations. Studies have shown that the majority of PD patients have non-motor symptoms that do not respond to levodopa treatment and therefore may not achieve meaningful relief with dopaminergic neuron replacement strategies [40]. Fetal ventral midbrain (VM) transplantation trials initiated at Lund University in the late 1980s reported significant improvements in motor symptoms but no benefit in non-motor manifestations, confirming this limitation [41]. Very recently, a phase I/II trial of iPSC-derived, CORIN-sorted dopaminergic cells for PD was completed (The Kyoto Trial) [24,41]. Similarly, no improvement in non-motor symptoms was observed in this open-label trial, as assessed by the Movement Disorder Society-sponsored revision of the Unified Parkinson‘s Disease Rating Scale (MDS-UPDRS) Part I [24,42].

**Figure 1:**
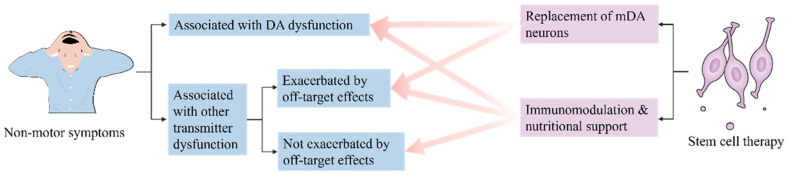
Non-motor symptoms that mDA neuron replacement strategies may be able to treat. The mDA neuron replacement strategy can treat non-motor symptoms associated with dopaminergic system dysfunction and non-motor symptoms exacerbated by off-target effects. Non-motor symptoms caused by functional impairments outside the dopaminergic system that are not related to drug-related off-target effects may only be ameliorated by neuroprotective strategies such as immunomodulation and nutritional support.

Some evidence suggests that dopaminergic neuron transplantation can actually improve a portion of non-motor disorders (Figure 1) [43]. For example, after transplanting human ventral mesencephalic (hVM) grafts into the dorsal striatum of a rat model of 6-hydroxydopamine (6-OHDA) lesions, Lelos et al. found that non-motor deficits associated with dopaminergic dysfunction, specifically visuospatial function and motivational processing, were improved [44]. In addition, some studies have reported that autologous transplantation not only confers immunological advantages but also alleviates depressive symptoms, making it a potential adjunctive approach for managing neuropsychiatric symptoms in PD [45]. However, the improvement in non-motor symptoms with stem cell therapy was limited to symptoms associated with DA loss, and it did not demonstrate improvement in symptoms associated with other neurotransmitter dysfunctions. Nevertheless, increasing evidence indicates that DA signaling is highly heterogeneous across cell types, circuits, and temporal scales, and that subtle dysfunctions in DA dynamics—rather than complete neuronal loss—may preferentially impair cognitive, learning-related, and motivational processes in early or prodromal PD [46]. From this perspective, stem cell-based dopaminergic interventions may exert partial benefits on non-motor symptoms by modulating disrupted dopaminergic signaling patterns or restoring aspects of physiological DA dynamics, even if they do not broadly normalize non-dopaminergic systems. There have also been studies documenting the clinical outcomes of two PD patients treated with autologous fat-derived stromal vascular fraction (SVF) cell preparations implanted in the face and nasal cavity. The researchers found that non-motor symptoms improved in both patients [47], but the main role of SVF in vivo may be the secretion of a variety of paracrine factors with immunomodulatory and trophic activities, including anti-inflammatory, anti-fibrotic, anti-apoptotic and angiogenic properties instead of the replacement of dopaminergic neurons [48,49]. It is different from what we often refer to as classical stem cell therapy, but it also suggests that immune modulation and nutritional support may have a role in improving non-motor symptoms. Finally, it has also been suggested that the primary effect of dopaminergic cell replacement on non-motor symptom control may only be fully realized after long-term follow-up [50], but this has not yet been supported by research. Although all of these studies point to aspects of stem cell therapy that may improve non-motor symptoms, it is unlikely that stem cell therapies that focus on dopaminergic neuron replacement will resolve most of these non-motor symptoms which are not due to dopaminergic pathology and therefore will not be able to completely “cure” PD [51]. The specific mechanisms underlying the improvement in non-motor symptoms observed in past studies remain to be elucidated. What is clear to us is that the mechanisms described above that cause non-motor symptoms imply that the core of non-motor symptom treatment in PD may not lie in the replacement of dopaminergic neurons, but rather in the restoration of important molecular functions.

### 2.2. Genetic Defects in Autologous Cells

PD can be viewed as a genetic disorder, as some patients harbor well-defined rare pathogenic variants that give rise to familial forms of the disease, while in many others disease susceptibility reflects the combined effects of multiple common genetic risk variants [52]. Starting with the landmark discovery of synaptic nucleoprotein-alpha (SNCA) mutations in 1997 [53], there are now more than 100 different genes or loci that have been clearly associated with susceptibility to PD [54,55,56,57]. Among these, several genes, including LRRK2, PRKN (PARK2), PINK1, DJ-1, and VPS35, have been widely reported to cause familial PD through dopaminergic neurodegeneration [58,59,60]. In addition to these pathogenic variants with high penetrance in PD genes, a large number of common variants with relatively small individual effects contribute in aggregate to an increased genetic susceptibility to sporadic (so-called “idiopathic”) PD [53,61]. This highlights that genetic factors, as important contributors to PD, play a role in both sporadic and familial forms of the disease through complex mechanisms [62,63,64]. A considerable fraction of patients with PD, up to about 20%, report a family history of the condition [65]. At the population level, based on twin studies and statistical genetic approaches, the heritability of PD has been estimated to range between 22% and 40% [52]. At the individual level, identifiable pathogenic variants are detected in approximately 5–15% of patients [66], and this proportion can be substantially higher in certain populations, such as those with early-onset PD, in whom it may exceed 40–50% [67,68].

Cells derived from the patient themselves do not require long-term immunosuppressive therapy after transplantation, thereby avoiding immune rejection and immunosuppression-related complications, which may confer a more favorable overall risk–benefit profile compared with allogeneic or human leukocyte antigen (HLA)-matched transplantation strategies [69]. However, a major limitation of autologous cell-based approaches is that autologous iPSC-derived mDA neurons may retain pathogenic germline or somatic variants present in the donor, thereby increasing their vulnerability and potentially reducing therapeutic efficacy [70,71,72]. Bieri et al. found increased aggregation of α-syn in mouse primary neurons expressing the LRRK2 G2019S mutation associated with PD. To validate this finding in a human setting, they established a novel human α-syn transmission model using iPS-derived neurons (iNs), in which human α-syn preformed fibrils (PFFs) triggered endogenous α-syn aggregation in a time-dependent manner. In PD subject-derived iNs, the G2019S mutation enhanced α-syn aggregation, whereas LRRK2 deletion reduced aggregation [73]. Another study also found that PD patient-specific iPSC-derived mDA neurons recapitulated PD phenotypes, including pathogenic protein accumulation, cell type-specific vulnerability, mitochondrial dysfunction, and neurotransmitter homeostasis abnormalities [74]. Devine et al. produced multiple iPSC lines from an SNCA triplication patient and an unaffected first-degree relative. When these cells differentiated into midbrain dopaminergic neurons, cells from the patient produced twice the amount of α-syn as neurons from the unaffected relative [75]. These findings indicate that patient-derived mDA neurons exhibit cellular dysfunctions relevant to PD pathophysiology. Notably, disease-associated cellular abnormalities, including defects in mitosis and autophagy, as well as epigenomic and transcriptomic alterations, have been identified in dopaminergic neurons derived from patients with sporadic PD, reflecting the influence of complex, non-monogenic genetic backgrounds rather than single mutations [76,77,78]. Together, these observations underscore that autologous iPSC-derived dopaminergic neurons may retain not only rare causal mutations but also polygenic risk architectures that predispose them to disease-relevant cellular dysfunction [79]. Moreover, both hESCs and iPSCs inevitably accumulate genomic aberrations during expansion, which remains a significant concern for their clinical application [80].

Nevertheless, it has been proposed that, because PD typically manifests only after decades of preserved dopaminergic neuron function—even in rare and severe genetic forms—it is reasonable to assume that transplanted young dopaminergic neurons would remain functionally adaptive and capable of effective synaptic signaling for many years [69]. Some animal studies have suggested that iPSC-derived mDA neurons from patients with PD do not differ significantly from those derived from healthy donors in terms of functional recovery, and they are similarly capable of reconstructing dopaminergic synapses and improving motor phenotypes [81,82]. The necessity of genetic correction of autologous cells remains to be further investigated, but it is undeniable that the presence of genetic defects in autologous cells represents a potential limitation and risk for stem cell-based therapies in PD.

Mitochondrial DNA (mtDNA), as a non-negligible genetic factor, also participates in the pathogenesis of PD. Genetic alterations affecting mtDNA maintenance, exemplified by POLG mutations, are well-established causes of PD [83]. MtDNA itself harbors pathogenic variants and age-related somatic mutations that may contribute to both familial and sporadic PD. For instance, elevated levels of mtDNA deletions have been observed in dopaminergic neurons of the substantia nigra in patients with sporadic PD, and they are closely associated with mitochondrial respiratory defects and impaired energy metabolism, which may increase neuronal vulnerability [84]. Notably, mtDNA mutations may provide a plausible genetic explanation for a subset of patients with PD in whom no definitive pathogenic variants in nuclear genes are detected. Therefore, we cannot ignore mutations in the mitochondrial genetic material carried by autologous cells either.

In addition to disease-related mutations inherently present in patient-derived cells, iPSCs also carry a risk of acquiring de novo mutations during somatic cell reprogramming, such as C→T transitions at CpG sites, which may be associated with large-scale DNA demethylation and epigenetic remodeling intrinsic to the reprogramming process [85,86]. As this issue primarily reflects a technical challenge in the safety and quality control of iPSC reprogramming rather than an inherent limitation of stem cell-based therapies themselves, it is not discussed in detail in this review.

### 2.3. Unimproved Pathogenesis

Initial post-transplant studies found no graft pathology within the first 10 years; however, LBs began to appear in donor-derived neurons 11–16 years after transplantation, and the proportion of affected donor cells increased in patients surviving >20 years [87]. LBs containing α-syn were found in approximately 10–15% of donor cell-derived dopaminergic neurons in postmortem tissues from patients who survived more than 20 years after the transplantation of fVMs [88,89,90]. Although LBs were detected in only 10–15% of donor-derived neurons, soluble α-syn was elevated in up to 80% of donor cell somata, a change consistent with premature cellular senescence [50]. Subsequent studies have shown a gradual decrease in DA transporter and tyrosine hydroxylase expression levels in some transplanted neurons over time, further supporting the idea that the disease process directly affects the transplanted cells [50,91]. Since the transplanted cells are derived from fetal tissue and are unlikely to have a predisposition to PD, this LB pathology suggests that the graft may be affected by the host disease [92,93].

α-Syn is a membrane-bound protein found in presynaptic terminals and is critically involved in the regulation of synaptic function and plasticity [94]. In normal conditions, α-syn exists as a soluble, intrinsically unstructured monomer, but under pathological conditions it can misfold and aggregate into oligomers, ultimately forming insoluble inclusions that constitute the major components of LBs and Lewy neurites [95,96]. It is now believed to contribute substantially to dopaminergic neurodegeneration [97]. α-Syn seeding activity refers to the ability of misfolded α-syn aggregates to induce the misfolding of normal α-syn molecules in vitro [98,99,100]. Studies have demonstrated that α-syn aggregates can spread in vivo in a prion-like manner through three major mechanisms—exosome-mediated intercellular transport [101,102], bidirectional transfer via tunnelling nanotubes between neurons and glial cells [103,104], and receptor-mediated uptake involving specific α-syn fibril receptors [105]—thereby providing plausible routes by which host pathology may be transferred to transplanted donor cells. In addition, impaired α-syn clearance by host astrocytes may further aggravate the pathological microenvironment surrounding transplanted cells [106].

Different studies have given conflicting accounts of the effects of α-syn on donor cells. Some researchers have argued that despite this host pathology phenotype in some transplanted cells, the survival and function of the transplanted cells appear to be relatively unaffected, and that fetal tissue transplantation can provide long-term therapeutic benefits [51]. For example, Kefalopoulou et al. found that transplanted neurons continued to exert positive functional effects despite being affected by the patient’s disease process [50]. At the same time, some studies suggest that under neurodegenerative pathological conditions, glial cells can transfer mitochondria to neurons as a compensatory mechanism, which may help partially restore mitochondrial function and metabolic homeostasis in transplanted cells affected by the host disease pathology [107]. However, most studies still support the view that the presence of endogenous pathological proteins may affect the transplanted functional cells, causing them to show pathological changes similar to PD, and therefore the therapeutic effect may not be maintained for a satisfactory period of time [108,109,110]. It has been shown that the burden of LBs in grafts is associated with a reduction in symptomatic benefit for patients [111]. α-syn aggregates not only propagate between neurons but also exert direct toxicity on dopaminergic neurons, leading to cellular dysfunction, including mitochondrial impairment, endoplasmic reticulum stress, disruption of autophagy-lysosomal pathways, and synaptic and nuclear dysfunctions [112,113], and they additionally act on glial cells to trigger neuroinflammatory responses [114]. As mentioned above, post-mortem analyses of patients who survived for more than 20 years after transplantation revealed elevated levels of soluble α-syn in up to 80% of donor cell bodies, indicating that the influence of host α-syn pathology on transplanted cells should not be underestimated. Emerging multiomic studies indicate that dopaminergic neurons display heterogeneous responses to PD pathology, implying that transplanted neurons of different origins or differentiation states may be differentially influenced by the host microenvironment, leading to variable therapeutic outcomes and adding further complexity to cell-based therapies [115]. Moreover, differences in the susceptibility of dopaminergic neurons to PD pathology arise from procedural differences in transplantation methods [116,117]. When fetal tissue is dissociated into a single-cell suspension prior to transplantation, the resulting grafts exhibit none or only very few α-syn inclusions even 14 years after transplantation [118,119]. In contrast, when cellular aggregates rather than dissociated cells are transplanted, activated microglia are frequently observed surrounding the grafts [93,120], and approximately 2–12% of dopaminergic neurons within the grafts contain α-syn–positive inclusions 12–24 years after transplantation [92,120,121]. The long-term effects of α-syn on the efficacy of stem cell therapy need to be further investigated. Overall, the current cell replacement approaches are expected to improve symptoms by restoring dopaminergic neurons, but they do not eliminate the underlying α-syn pathology and therefore are unlikely to halt disease progression [41]. Notably, most preclinical assessments rely on the 6-OHDA rodent model or the 1-methyl-4-phenyl-1,2,3,6-tetrahydropyridine (MPTP) primate model, both of which lack endogenous α-syn pathology. As a result, these models do not recapitulate the toxic environment present in the patient’s brain—characterized by α-syn accumulation, its propagation, and inflammation—into which donor cells are ultimately transplanted. This discrepancy highlights that clinical grafts inevitably face a far more hostile microenvironment than predicted by animal studies [71].

In addition to the effects of α-syn, the overall pathological microenvironment of the PD brain, such as neuroinflammation and immune dysregulation, iron homeostasis imbalance, and mitochondrial dysfunction, also impedes the normal survival, differentiation, maturation, and function of transplanted cells. Growing evidence indicates that patients with PD exhibit chronic neuroinflammation, characterized by activated microglia [122,123,124], reactive astrocytes [125], and infiltrating T cells [126] within the central nervous system. This heightened inflammatory state likely exacerbates the vulnerability of transplanted cells, further compromising their survival, integration, and long-term functional stability [114,127]. Injuries occurring during cell transplantation may also alter the patient’s own brain microenvironment or even cause the host brain to become hostile to the transplanted cells after transplantation, thus impairing the normal physiological function of some of the transplanted cells and greatly decreasing the efficiency of transplantation. In addition, accumulating evidence indicates that excessive iron accumulation occurs in SNc of patients with PD, accompanied by dysregulation of iron transport systems. This disruption of iron homeostasis promotes oxidative stress and lipid peroxidation, which can induce ferroptosis in dopaminergic neurons, thereby further impairing the survival and functional integration of transplanted cells [128,129]. As discussed above, intercellular mitochondrial transfer from glial cells to neurons may act as a compensatory mechanism in PD; however, the transferred mitochondria may themselves be functionally impaired, which may exacerbate mitochondrial dysfunction and functional disturbances in transplanted neurons [107]. Metabolic disturbances and glycemic variability coexisting in patients with PD may interact with PD-related pathological processes, contributing to an unfavorable host microenvironment and potentially affecting the survival and functional stability of transplanted donor cells [130]. Other factors that may influence the survival and functional performance of transplanted neurons include neuromelanin accumulation in the substantia nigra [131], excessive generation of reactive oxygen species (ROS), and impaired antioxidant defenses [132,133].

### 2.4. Difficult-to-Establish Neural Circuits

Ideal transplantation targets for stem cell therapy have not yet been systematically identified [51]. In cellular therapies for PD, donor dopaminergic neural progenitor cells are usually injected into the putamen to form and function in local neural circuits with host neurons [23,134,135]. However, the putamen is the projection field of A9 (substantia nigra pars compacta) dopaminergic neurons, rather than the location of their cell bodies [51]. The rationale for this approach is the concern that axons from grafts implanted in the substantia nigra might not extend far enough to reinnervate the human striatum [87]. Attempts have been made to transplant dopaminergic neural progenitor cells into the substantia nigra via orthotopic transplantation [136,137]. However, although some surviving donor neurons in the substantia nigra extend neurons into the putamen, the functional benefits are less than those of ectopic transplantation [138]. Furthermore, in some studies, dopaminergic neurons transplanted into the midbrain did not survive as well compared to the striatum [139]. Also, transplantation studies over the past decade have shown that intra-striatal transplantation of fetal tissue is sufficient to restore striatal DA release and induce recovery from Parkinson symptoms in at least some patients [50].

**Figure 2:**
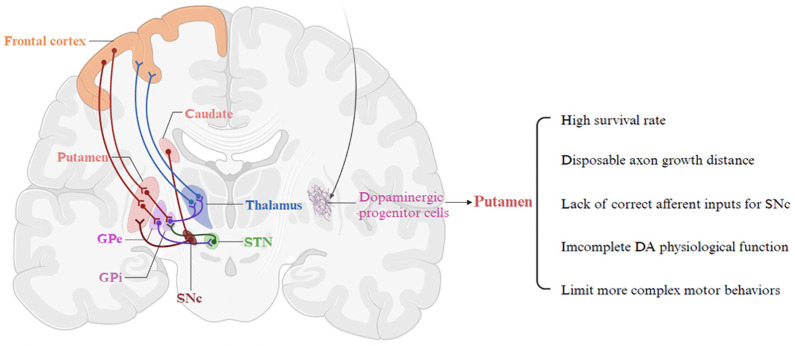
Normal neural circuits and characteristics of ectopic transplantation. The left side of the figure shows the normal neural circuitry of the basal ganglia system. It can be seen that mDA neurons located in the SNc also receive neural afferents from sites such as the caudate nucleus. Consequently, conventional ectopic transplantation, i.e., transplantation of stem cells into putamen, while shortening the distance of axonal growth and improving cell survival, lacks the correct afferent inputs to the mDA neurons, disrupts the integrity of dopaminergic physiology, and prevents the restoration of more complex motor functions compared to orthotopic transplantation.

However, ectopic transplantation raises the concern that the grafted cells may lack the major afferent inputs from endogenous mDA neurons in striatal neuronal activity. It has been found that presynaptic inputs are location-dependent, i.e., human mDA neurons grafted to the striatum and substantia nigra receive inputs mainly from different brain regions [140]. Human mDA neurons transplanted into the substantia nigra receive a wide range of inputs from similar brain regions as endogenous mDA neurons, whereas mDA neurons transplanted into the striatum receive inputs from different sources [141]. And it is known that these inputs play an important role in the phasic regulation of nigrostriatal neuronal activity. Lack of normal afferent control may limit the cell’s ability to improve more complex motor behaviors [142]. Moreover, there is growing evidence that DA released from substantia nigra (SN) localized dendrites may have important physiological functions distinct from striatal DA release [143]. Thus, ectopic transplantation may prevent donor cells from establishing normal and effective neural afferents, making it difficult to improve some complex motor behaviors (Figure 2). Beyond the inability to establish appropriate neural circuitry, ectopic transplantation may also render grafted dopaminergic neurons more vulnerable to α-syn pathology. Increasing evidence indicates that the transplantation site critically influences host-to-graft α-syn transmission. In mouse models of PD, dopaminergic neurons grafted into the striatum exhibit a higher accumulation of host-derived α-syn than those transplanted orthotopically into the substantia nigra, although no significant difference is observed in the levels of pathological phosphorylated α-syn (pSer129) [144]. These findings suggest that the substantia nigra may provide a more permissive microenvironment for grafted neurons, characterized by reduced α-syn propagation and potentially improved graft stability.

In addition, Cochen et al. found that ligand binding of DA transporters in the cerebral cortex was unchanged despite a significant increase in 18F dopa uptake in patients after transplantation. This finding suggests that the clinical benefit induced by transplantation is related more to an increase in dopaminergic activity than to an improvement in dopaminergic innervation in the host striatum [145]. This could mean that the main role of stem cell transplantation is still at the level of DA supplementation.

### 2.5. Limitations of Disease Parameters and the Patient’s Condition

Current studies on stem cell transplantation for PD indicate that both the magnitude and durability of therapeutic benefit vary substantially among individual patients [146]. Reports and experiences from previous clinical studies of stem cell therapy help determine which patients may benefit from cell therapy [138]. These studies suggest that the onset and progression of PD itself, including age of onset, staging, duration, and subtypes, may be able to predict clinical outcomes [147]. It has been suggested that the successful clinical outcome of cell replacement therapy depends on the integrity of the nigrostriatal dopaminergic system of the recipient [148]. Appropriate reinnervation of the host striatum may play a key role in functional recovery in PD, and therefore the recipient’s nigrostriatal dopaminergic system should be adequately functional prior to transplantation [109]. An early study by Anders Bjorklund and colleagues reported that the functional improvement resulting from fVM cell transplantation depends largely on the severity of damage to the host nigrostriatal system. Functional recovery in rats that lost more than 70% of their dopaminergic innervation was superior to that in rats with complete damage to the dopaminergic system, suggesting that remnants of the host dopaminergic system may be necessary for grafts to have a functional effect [149]. Barker et al. observed a high survival rate of transplanted cells in some patients without beneficial improvements in behavior. This may indicate the degeneration of other brain systems in this group of patients, particularly the postsynaptic components of the dopaminergic system [150,151,152,153]. It can be seen that stem cell therapy is not promising for patients with PD in more advanced stages or with longer and more severe disease progression [152,154], when neurons in the striatum and cortex have degenerated to the point where they are no longer responsive to DA secreted by the transplanted neurons. If a patient remains responsive to levodopa—indicating preserved postsynaptic dopaminergic signaling—they may be considered a favorable candidate for cell therapy [155]. Rapid disease progression in non-dopaminergic systems may also compromise the therapeutic efficacy of stem cell-based interventions. The recent phase I/II trial of iPSC-derived cells (the Kyoto Trial) showed that two patients did not exhibit motor improvement, which may be attributable to their relatively rapid disease progression, particularly within non-dopaminergic systems [24,42].

It has also been suggested that age of onset and stage and duration of PD have variable predictive effects on different aspects of efficacy [42]. The researchers conducted a systematic statistical analysis of the clinical outcomes of PD patients who received fVM transplants and compared them with various parameters, namely, age of onset (advanced, greater than 40 years, versus young, less than 40 years), disease stage (severe versus mild), and duration of the disease (long duration, greater than 10 years, versus short duration, less than 10 years). The results found that graft survival was not related to age at onset, but it was related to disease stage and duration. Transplant survival is higher in patients with mild PD and shorter disease duration (≤10 years). Also, the researchers tested various factors of clinical improvement after transplantation in patients with PD using the Unified Parkinson’s Disease Rating Scale (UPDRS) motor score. The results showed that PD patients who underwent fVM transplantation all showed significant clinical improvement after transplantation. Meanwhile, comparisons between the parameters (i.e., old vs. young, severe vs. mild, and long vs. short) after transplantation did not show significant differences, i.e., none of these three factors were determinants of clinical improvement after transplantation [7,153]. Nonetheless, it was possible to find that patients with mild PD of short duration had the best outcomes in most of the studies.

Additionally, different PD subtypes may also influence the response to stem cell therapy. Although direct evidence from stem cell transplantation trials stratified by PD subtype remains limited, available data suggest that tremor-dominant (TD) patients, who generally show slower progression, less widespread pathology, and better outcomes with circuit-based therapies, may be more likely to maintain symptomatic benefit after dopaminergic cell replacement, whereas akinetic-rigid, postural instability and gait difficulty (PIGD), and other non-tremor-dominant (nTD) subtypes, which are associated with faster progression, greater cognitive and axial burden, and more extensive non-dopaminergic involvement, may derive less durable benefit from graft-based strategies [156,157,158]. The differential efficacy of stem cell therapy across PD subtypes still requires further investigation.

In addition to the parameters associated with PD, the patient’s own age and gender are also factors that influence the therapeutic outcome of stem cell therapy. Studies have shown that younger patients tend to have a better prognosis after stem cell transplantation [159], whereas age greatly affects the survival of dopaminergic neurons due to aging-induced blood–brain barrier (BBB) leakage that may increase neuroinflammation and heighten vulnerability to peripheral insults [7], as well as lower brain plasticity in older patients [22]. In 2001, Freed and colleagues conducted the first double-blind study of 19 patients with PD, comparing the effects of fetal neural stem cell (fNSC) transplantation in patients younger than 60 years of age to those older than 60 years of age, and the results of the study showed that younger patients with PD had a more pronounced improvement in motor symptoms than older patients [135]. They also found that while the survival of transplanted embryonic dopaminergic neurons in the thalamus of PD patients was independent of age, the transplants had some benefit only in patients 60 years of age and younger, but not in older patients [22]. This suggests that the host brain microenvironment may play an important role in the therapeutic efficacy of transplanted neural stem cells [160]. Moreover, because iPSCs derived from elderly donors often exhibit reduced differentiation competence, autologous iPSC-based approaches may result in suboptimal therapeutic outcomes in older patients [161]. However, one study with ongoing follow-up of patients found that there may be no significant difference in outcome between patients of different ages after 1 year of transplantation [159]. There are also studies that did not find a selective benefit in younger patients, which may of course be related to the small number of patients participating in the trials [23]. Regarding the gender of the patient, studies have shown that PD imposes a greater disease burden on men than on women [162], while women may respond more favorably to treatment [163]. The protective effects of estrogen, greater striatal dopaminergic activity in women, and higher levodopa availability may account for the sex differences in disease burden and treatment response [163,164,165]. For example, in a randomized controlled trial, the proportion of female survivors was higher [166]. However, some studies have found that men have relatively better clinical improvement at 1 year compared to female patients, but similar to the age factor, the relationship between clinical outcomes and gender may not persist long after 1 year [159].

**Figure 3:**
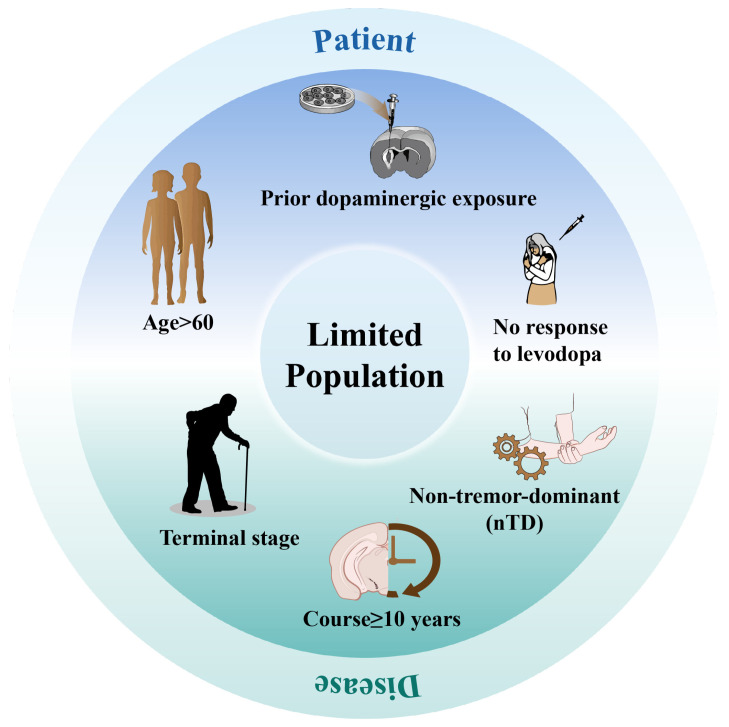
Limited population of stem cell therapy. The population unsuitable for stem cell therapy can be assessed from both the patient’s own parameters and the disease parameters. In terms of the patients, people who are older than 60 years of age, who have had prolonged prior DA exposure (e.g., long-term medication and stem cell transplantation), or who have not responded to levodopa therapy will have limited benefit from stem cell therapy. In terms of disease, if PD has progressed to an advanced stage or has been present for more than 10 years, then the role of stem cell therapy in improving the disease is also limited; in addition, nTD phenotypes may also represent a less favorable subgroup, although this possibility still requires further clinical validation.

Notably, a number of variables exacerbate the prognosis of the disease and complicate treatment. For example, a tremor-free presentation, rapid loss of independent limb function, a positive Babinski sign, and the presence of cognitive deficits in the first year are major adverse prognostic factors [147]. In addition, other factors such as long-term dopaminergic medication exposure may also affect the effectiveness of stem cell therapy. Studies have shown that stem cell-based PD therapies may not enable patients to achieve established treatment outcomes when taking medication for a long period of time or undergoing stem cell transplantation over a long period of time [152,167,168] (Figure 3).

## 3. Strategies and Future Directions

These limitations indicate that stem cell therapy alone is unlikely to fully meet the therapeutic needs of PD. In parallel with cell replacement strategies, a growing number of non-cell-based approaches are being explored for PD, including gene therapy, neuroprotective strategies, in vivo reprogramming, and immunomodulatory interventions targeting α-synuclein pathology. Compared with stem cell transplantation, these approaches may offer advantages in modifying disease progression without the challenges of graft survival and circuit integration [169,170]. However, they also face limitations, including delivery and safety challenges for gene therapy [169], translational and trial-design barriers for neuroprotective strategies [171], preclinical immaturity and inconsistent efficacy for in vivo reprogramming [172], and uncertain clinical efficacy for α-synuclein-targeted immunotherapy [170]. Given that both stem cell therapy and in vivo reprogramming can provide new dopaminergic neurons within the patient, the respective advantages and limitations of these two approaches are summarized in Table 2. Accordingly, stem cell therapy should be viewed as part of a broader therapeutic landscape, where combination strategies may ultimately provide the most effective intervention. In addition, orthotopic transplantation and more precise patient stratification may also represent promising strategies for further improving therapeutic outcomes.

**Table 2:** Comparison between stem cell therapy and in vivo reprogramming for Parkinson’s disease.

	Stem Cell Therapy	In Vivo Reprogramming
Basic principle	Replacement of lost DA neurons by transplantation of exogenous stem cells or stem cell-derived dopaminergic progenitors/neurons.	Direct conversion of endogenous brain cells, particularly glial cells, into dopaminergic-like neurons in situ, without ex vivo cell preparation or transplantation [172,173].
Need for transplantation	Required.	Not required [172,173].
Cell source	Fetal ventral mesencephalic tissue, hESCs, iPSCs, and related cell products.	Endogenous brain cells, especially astrocytes [172,173].
Clinical maturity	Supported by a more mature evidence base; fVM-, hESC-, and iPSC-based approaches have entered human clinical trials.	Still largely confined to animal studies and remains far from clinical application [172,174].
Evidence of functional recovery	Substantial evidence supports long-term graft survival, reconstruction of striatal dopaminergic innervation, and sustained motor improvement in some patients and animal models.	Proof-of-concept studies have shown generation of functional DA-like neurons in PD mouse brains with partial motor improvement, although consistency across studies remains limited [172,173].
Immune rejection/immunosuppression	Allogeneic transplantation is associated with immune-related concerns; autologous iPSC-based approaches may partially mitigate this issue.	In vivo reprogramming may avoid or substantially reduce the need for immunosuppression and the risk of immune rejection [172,173].
Tumorigenic risk	Particularly relevant for iPSC/hESC-based products because of pluripotency and residual undifferentiated cells, requiring stringent purification and long-term safety evaluation.	In vivo reprogramming may reduce or avoid tumorigenic risk associated with passage through a pluripotent stage [175,176].
Circuit integration	Evidence indicates that grafted cells can form synaptic connections, receive host inputs, and achieve functional integration; however, most studies still rely on ectopic transplantation.	In vivo reprogramming has the potential to integrate directly into local circuits in situ, but its projection pattern and long-term functional integration remain debated [172].
Influence of the host pathological microenvironment	Transplanted cells remain exposed to the pathological microenvironment of PD.	In vivo reprogramming may have disease-modifying potential by converting reactive glia and reducing harmful glial responses, although current support is mainly theoretical or based on early evidence [172].
Patient-specific genetic defects	Autologous iPSC-derived cells may retain disease-causing mutations, sporadic genetic susceptibility, and mitochondrial abnormalities present in the donor.	Likewise, it cannot inherently eliminate the patient’s original genetic background or pathological susceptibility [172].
Standardization and scalability	hESC-derived products have potential for GMP-compatible production, storage, and quality control, whereas individualized iPSC preparation remains costly and time-consuming.	The workflow is theoretically simpler because it avoids ex vivo expansion, differentiation, and transplantation, but reprogramming efficiency, uniformity, and reproducibility remain major challenges [172,176].
Controllability of cell identity and quality	In vitro differentiation, sorting, and quality control are relatively easier to implement before transplantation.	After direct in vivo conversion, cell identity, purity control, and long-term phenotypic stability are more difficult to define and monitor [172].

### 3.1. Orthotopic Transplantation

For the possible adverse consequences of ectopic transplantation, we should perhaps consider transplanting stem cells in situ. Cardoso et al. transplanted VM-patterned hESC-derived progenitor cells into the midbrain of 6-OHDA-lesioned rats and used a rabies virus-based monosynaptic tracing technique to reveal the source and extent of presynaptic inputs from the host to the grafts. The results showed that the transplanted neurons had the ability to gradually extend axonal projections to appropriate forebrain target structures over 24 weeks and that the timing and extent of innervation of graft-derived dopaminergic fibers to the dorsolateral striatum matched the amphetamine-induced reduction of rotational asymmetry in the animals. This suggests that in situ transplanted mDA cells can still innervate downstream neurons correctly and function accordingly. Also, using a retrograde, monosynaptic tracing technique based on pseudorabies virus, they demonstrated that the host establishes afferent inputs to the transplanted cells from 6 weeks after transplantation and that a recovery of motor function can be observed in these animals. This phenomenon, in turn, suggests that the transplantation of mDA progenitor cells in situ allows mDA cells to establish normal afferent inputs to upstream neurons [177]. Despite promising results in animal experiments with orthotopic transplantation, behavioral readouts in nigrostriatal transplantation studies are usually limited to drug-induced rotational tests, which do not rely on afferent inputs to the grafts [178], i.e., it is unknown exactly what role afferent inputs play in behavioral improvement. Thus, testing whether intranigral transplantation triggers rescue of more complex motor behaviors and whether these behaviors are dependent on afferent inputs or release of nigral DA remains to be investigated further. In the meantime, it has been suggested that the human brain is much larger than that of rodents and rhesus monkeys, and that if neurons are transplanted into the SN, the length of neuronal projections may still be insufficient to innervate the striatum [179]. However, the ability of human pluripotent stem cell (hPSC)-derived neurons to extend axons far enough to re-innervate the human brain has been demonstrated in nonhuman primates by intraspinal transplantation experiments [82,180]. In addition to hPSC, hESC-derived VM progenitor cells can also extend axons over long distances to re-innervate distant forebrain targets [181,182].

### 3.2. Combining with Gene Editing, Small Interfering RNA, and Other Technologies to Regulate the Expression of Specific Genes

When using autologous cells for transplantation, we can target pathogenicity gene mutations that the autologous cells themselves may carry. Mutations in several genes, including SNCA (such as A30P and A53T), PARK2 (Parkin), DJ-1 (PARK7), PINK1, and LRRK2, have been linked to genetic forms of PD [183]. Gene therapy by introducing RNA interference (RNAi) approaches, such as small interfering RNAs (siRNAs) or short hairpin RNAs (shRNAs) targeting mutated genes or ectopic expression of relevant regulatory genes (e.g., DA biosynthetic enzymes), may help reduce cytotoxicity [109]; however, efficient delivery and long-term stability of RNAi in grafted neurons remain technical challenges. To prevent the pathological microenvironment of PD from affecting the donor cells, we can also use gene therapy to improve the donor cells and increase the resistance of normal cells to α-syn. Previous studies have shown that endogenous soluble α-syn in the cytoplasm is necessary for the formation of LBs and toxic effects on mDA neurons [184]. If neurons lack endogenous expression of α-syn, they are less susceptible to α-syn PFFs seeding [185]. Moreover, when SNCA^−/−^ mice were given stereotaxic injections of α-syn PFFs, unlike wild-type mice, they showed no signs of synucleinopathy or neurodegeneration [186]. Therefore, using CRISPR/Cas9n gene-editing to reduce SNCA (α-syn) expression in hPSCs may generate mDA neurons with increased resistance to a-syn-related pathology [187]. Such disease-resistant cells are particularly important in young PD patients or in patients with hereditary PD with a substantial α-syn burden or aggressive synucleinopathies, such as patients with SNCA-proliferative PD [188] and glucocerebrosidase (GBA) mutation carriers. Researchers have now used ESCs to grow human dopaminergic neurons that are resistant to synucleinopathy as follows: Chen et al. created SNCA^+/−^ and SNCA^−/−^ cell lines by SNCA gene KO in hESC cell lines using CRISPR/Cas9 technology and differentiated them into midbrain dopaminergic neurons. Subsequently, they were treated with recombinant α-syn-preformed fibrils to seed the formation for Lewy-like pathology. Wild-type neurons are susceptible to protein aggregation; in contrast, SNCA^+/−^ and SNCA^−/−^ cells show resistance to the formation of this pathological hallmark [187]. Gene therapy not only enhances the resistance of mDA progenitor cells to the pathological microenvironment, but it also achieves long-term stability of the therapeutic effect in the brain with a single intervention [189]. The lack of major dysfunction in SNCA KO mice further confirms the feasibility of this approach [190]. In addition to this, we can design gene therapy strategies from the perspective of the α-syn intercellular diffusion pathway. The mechanisms why the same pathological aggregates can infect different cells along different pathways and lead to diseases with different clinical features remain unclear [191]. Hudák and his team found that multiligand proteoglycans in neuronal membranes can endocytose α-syn fibers through both lipid-dependent and lectin-independent pathways [192]. Meanwhile, another study showed that cofilin1, an actin-binding protein, also promotes the uptake of α-syn aggregates in healthy neurons [193]. Tunneling nanotubes (TnTs) are a type of intercellular substance transport channel. Valdinocci et al. demonstrated that α-syn aggregates can be transported between cells via TnTs using mitochondria as carriers [194]. Therefore, knocking out these types of genes in donor cells by gene editing can effectively prevent the transmission of α-syn between cells.

### 3.3. Combining with Neuroprotective Strategies

Most of the non-motor symptoms of PD are caused by complex molecular defects in multiple regions of the nervous system [34]. Early protection of the function of individual transmitter systems to slow disease progression from the outset is critical. At the same time, after stem cell transplantation, we should also guarantee the survival, proliferation, and differentiation of the transplanted cells. The possible effects of the pathological microenvironment of PD on donor cells suggests that a way to improve the brain microenvironment of the host is an important topic to be addressed in the future. Neuroprotective strategies, such as applying the neuroprotective effects of neurotrophins, activating neuroprotective signaling pathways (e.g., Nrf2-Keap1 [195], PI3K/AKT/GSK3β [196]), and protecting neurons from regulated forms of programmed cell death (e.g., ferroptosis [197]), are considered to be prospective approaches to improve the brain microenvironment [70]. Combining these approaches with stem cell therapy may support donor cell survival and maintain neuronal function and brain homeostasis [70]. Recent work further supports the value of coupling stem cell-based approaches with microenvironment-targeted interventions. For example, glycyrrhizic acid combined with adipose-derived mesenchymal stem cells synergistically mitigates 1-methyl-4-phenylpyridinium (MPP^+^)/MPTP-induced neurotoxicity by modulating autophagy through the PI3K/AKT/HIF-1α pathway, illustrating how activating protective signaling cascades can enhance donor cell survival and functional restoration [198]; the glucagon-like peptide-1 receptor (GLP1R) agonist semaglutide suppresses C3^+^ reactive astrocytes and significantly enhances the survival, differentiation, and therapeutic efficacy of transplanted neural stem cells in PD models, underscoring the value of modulating hostile glial states to optimize graft outcomes [199]. During the formation of the brain microenvironment in PD, cytokines may perform different functions in different environments or stages [70], highlighting that therapeutic modulation must be carefully tailored to the specific context and timing. Therefore, we cannot ignore the importance of cytokines in neuroprotective strategies and should focus on how to fully utilize the beneficial effects of cytokines and neutralize their negative effects. Functionalized biomaterial scaffolds (e.g., bioactive matrix-coated nerve conduits) incorporated with neurotrophic or pro-survival factors can provide a more permissive microenvironment for transplanted cells [6]. For example, an immunomodulatory encapsulation platform has been developed to deliver human induced pluripotent stem cell–derived dopaminergic progenitors (hiPSC-DPs) within semi-permeable alginate microcapsules loaded with locally released tacrolimus [200]. Consistently, transplantation of human iPSC-derived dopaminergic progenitors within a neurotrophin-enriched collagen hydrogel markedly enhanced graft survival and dopaminergic maturation in the parkinsonian rat brain [201]. Such encapsulation-based microenvironmental support strategies offer a complementary strategy to improve the stability and therapeutic efficacy of stem cell transplantation for PD. In addition to optimizing the host microenvironment, donor cell transplantation can be further strengthened by combining it with genetically engineered mesenchymal stem cells (MSCs) that constitutively secrete neurotrophic or cytoprotective factors, thereby providing a sustained, localized trophic supply that overcomes the short half-life, poor brain penetration, and limited temporal coverage associated with direct protein administration [202]. Multiple studies have engineered MSCs to overexpress neurotrophic or cytoprotective factors such as BDNF [203], Nurr1 [204], CDNF [205], PSPN [206], HGF [207], VEGF [208], NTN [209], or GDN [210], all of which improved dopaminergic neuron survival and behavioral outcomes in PD models.

Many recent studies have identified neuroprotective strategies that can mitigate the impact of α-syn accumulation in the host brain on transplanted donor cells. Co-culture of MSCs with neurons was shown to promote family with sequence similarity 134, member B (FAM134B)-mediated endoplasmic reticulum autophagy (ER-phagy), thereby accelerating the removal of ER-accumulated α-syn. This enhancement of selective ER-phagy not only lowered intracellular α-syn levels but also conferred a pro-survival effect in cellular models of PD and exerted neuroprotective benefits in vivo [211]. Co-transplantation of astrocytes may also be employed. Astrocytes, the most abundant glial cells in the CNS, exert key neuroprotective actions through antioxidant activity, glutamate clearance, and removal of toxic lipids, and they also secrete neurotrophic and anti-inflammatory factors [212,213]. Recent studies have shown that ventral midbrain (VM) astrocytes mitigate α-syn pathology by inhibiting neuronal α-syn aggregation and transmission, disassembling extracellular aggregates, scavenging α-syn fibrils, and promoting neuronal autophagic clearance [214,215]. Although concerns have been raised that transplanted astrocytes may become pro-inflammatory [216], accumulating evidence indicates that cultured astrocytes generally do not undergo detrimental activation and instead support neurite outgrowth [217]. Building on these findings, co-grafting dopaminergic progenitors with rodent-derived astrocytes in PD models reduced inflammatory responses, improved mDA neuron engraftment, and alleviated α-syn pathology [214,218]. These results suggest that incorporating astrocytes into grafts may enhance donor cell survival by mitigating both host inflammation and α-syn–related toxicity [71]. In addition, stem cell-based therapies may be combined with interventions that target α-syn propagation [219]. For example, accumulating evidence has identified membrane receptors such as FAM171A2, APLP1, and LAG3 as critical mediators of α-syn uptake and intercellular propagation. Therapeutic strategies that block α-syn–receptor interactions—either by selectively inhibiting FAM171A2-mediated early monomer transmission or by disrupting the APLP1-LAG3 receptor complex involved in fibrillar α-syn internalization—may effectively attenuate the spread of α-syn pathology to non-pathological grafted neurons [96]. In parallel, therapeutic strategies targeting exosomes and other extracellular vesicles merit increasing attention [220].

### 3.4. Combining Stem Cell Therapy with α-Synuclein Immunotherapy

Another promising strategy is to combine stem cell therapy with α-synuclein-targeted immunotherapy. Although current α-synuclein immunotherapies have not yet demonstrated a clear ability to slow or alter disease progression in PD, they remain mechanistically attractive because pathological α-syn can spread from cell to cell in a prion-like manner, and pathological forms of extracellular α-syn are accessible to antibody-based clearance [170,221]. Therefore, in the context of stem cell therapy, immunotherapy could serve as a complementary strategy to reduce extracellular α-syn burden, limit host-to-graft transmission, and thereby help preserve graft function after transplantation [221,222].

Active immunization may be one way to achieve such long-term support. Peptide vaccines such as PD01A, PD03A, UB-312, and ACI-7104.056 are designed to induce endogenous antibodies against pathological α-syn species, and early clinical studies have generally shown acceptable safety and immunogenicity, although clear clinical efficacy has not yet been established [223,224,225]. From the perspective of stem cell therapy, active immunization could be used as a maintenance strategy to provide sustained immune surveillance against pathogenic α-syn after grafting, potentially lowering the long-term risk of host-to-graft pathological spread. However, this approach is still limited by interindividual immune heterogeneity, the uncertain durability of therapeutic benefit, and the fact that most available data remain focused on safety and target engagement rather than proven disease modification [224].

Passive immunization offers a different and, in some respects, more controllable adjunct. Monoclonal antibodies such as prasinezumab and cinpanemab, as well as other antibody-based therapies, are intended to bind extracellular α-syn and reduce its aggregation, propagation, and toxicity; preclinical studies further suggest that such antibodies can attenuate neuropathology and prevent cell-to-cell transmission [222]. In a transplantation setting, passive immunization may be especially useful during the peri-transplant and early post-transplant periods, when donor cells are first exposed to the host microenvironment, because dosing can be titrated and treatment can be discontinued if adverse effects occur [221]. Nevertheless, passive immunotherapy also has important limitations, including the need for repeated administration, restricted brain penetration across the blood–brain barrier, and the fact that clinical trials to date have yielded mixed or inconclusive efficacy results [221,223].

In the future, combination strategies integrating stem cell therapy with immunotherapy could also be individualized on the basis of biomarkers, with patients showing stronger synuclein burden or more aggressive synucleinopathy potentially being preferential candidates for graft-plus-immunotherapy regimens [226]. Overall, although direct evidence for stem cell transplantation combined with α-syn immunotherapy is still lacking, the available literature provides a strong conceptual rationale for testing this strategy as a means to enhance graft durability and to overcome the hostile pathological microenvironment in PD.

### 3.5. Adjunctive Physical and Cognitive Training

Given that ectopic transplantation limits the establishment of appropriate afferent inputs and restricts the refinement of complex neural circuits, adjunctive physical and cognitive training has emerged as a promising strategy to enhance graft–host functional connectivity. Increasing evidence indicates that neural circuit maturation and synaptic refinement are critically dependent on activity-dependent mechanisms, both during development and during recovery from neural injury. Physical activity such as treadmill training transiently enhances neuroplasticity, promotes synaptogenesis, improves mitochondrial function, reduces inflammation, and increases neurotrophic factor secretion [227]. Importantly, activity-dependent cues stabilize active synaptic inputs and eliminate inactive ones, thereby fine-tuning graft-derived connections toward more physiological and functionally effective circuit architecture [227]. Consistent with this concept, combining dopaminergic neuron transplantation with structured physical training has been shown to enhance graft survival, axonal maturation, and synaptic connectivity in models of spinal cord injury and PD [228,229]. In a recent nonhuman primate study integrating treadmill exercise and cognitive tasks with hiPSC-derived dopaminergic neuron grafts, animals exhibited significant improvements in sensorimotor and cognitive functions, and histological analyses confirmed enhanced synapse formation between grafted and host neurons six months post-transplantation [230]. In a unilateral 6-OHDA rat model, optogenetic stimulation of grafted dopaminergic neurons produced sustained motor improvement only when stimulation was temporally coupled to goal-directed behavior, whereas stimulation delivered in the absence of behavioral engagement resulted in transient or unstable effects, underscoring the critical role of behavioral context in shaping graft-mediated functional outcomes [231]. These findings resonate with earlier evidence that adjunctive behavioral training facilitates the functional integration of transplanted neural tissues in models of Huntington disease and spinal cord injury [232,233]. Clinically, PD patients benefit from multiple modalities of physical activity—including treadmill running, cycling, yoga, tai chi, dance, dual-task training, and robot-assisted gait therapy—which can transiently improve stride length, cadence, postural stability, motor automaticity, cognitive flexibility, and procedural learning [234]. Given that the striatum plays a central role in habit learning and motor skill acquisition, newly established dopaminergic inputs from transplanted cells may further potentiate the benefits of physical rehabilitation, rendering the functional gains more robust and enduring [227]. Overall, these observations suggest that even optimally differentiated dopaminergic grafts may require structured, patient-tailored rehabilitation programs to fully integrate into host neural networks and overcome limitations imposed by ectopic transplantation. Designing future clinical trials to incorporate adjunctive physical and cognitive training—in accordance with WHO guidelines for moderate-to-vigorous intensity activity [235]—may therefore substantially enhance the efficacy and long-term durability of cell replacement therapies for PD. In addition to facilitating graft integration and neural circuit remodeling, accumulating clinical evidence indicates that exercise interventions significantly ameliorate non-motor symptoms in PD, particularly sleep disturbances and overall quality of life [236], and it may further improve the immunological microenvironment by attenuating chronic neuroinflammation, suppressing microglial and astrocytic reactivity, and modulating inflammasome- and cytokine-related signaling pathways, thereby creating a more permissive niche for graft survival and functional maturation [237].

### 3.6. Patient Stratification

There are likely specific indications and contraindications for cell therapy in PD. Many different factors can determine or influence the effectiveness of stem cell therapy. In future applications, identifying these confounding factors will help clinicians better stratify patients with PD and provide individualized treatment strategies for each patient accordingly [7]. Rather than aiming for a single best-practice or universally applicable intervention, an emerging view is that optimal care will depend on developing a diverse set of therapeutic options that can be matched to the needs of individual patients based on characteristics such as age at diagnosis, disease severity and duration, cognitive or autonomic dysfunction, and even socioeconomic considerations. The best outcomes for the broader PD population are therefore likely to be achieved through evidence-based treatment algorithms—similar to those already used in the pharmacological management of PD [238,239]—which help guide the allocation of appropriate interventions to specific patient subgroups. In this context, a realistic goal for stem cell-based strategies is to offer novel treatment modalities for those subgroups of patients who are most likely to benefit from long-term dopaminergic restoration and sustained improvements in motor function, ultimately translating into measurable gains in quality of life [240]. At present, several international preclinical research programs are underway [241,242] to uncover the mechanism of action of candidate stem cell lines, while phase I clinical trials are being conducted to determine the optimal parameters—such as patient age, disease duration, and symptom profile—to ensure that individual patients receive the most appropriate therapeutic intervention [243] (https://www.clinicaltrialsregister.eu/ (accessed on 13 April 2026); https://clinicaltrials.gov/ (accessed on 13 April 2026)). In addition to disease parameters and the patient’s condition, emerging PD biomarkers may also facilitate more precise patient selection. Recently, a number of new biomarkers for PD have been identified. For example, combining olfactory testing with a CSF α-syn seed amplification assay may facilitate the selection of a more well-defined group of patients with PD [98].

## 4. Conclusions

As mentioned earlier, despite the current limitations, combinatorial approaches and further mechanistic research may overcome existing barriers to clinical translation. At the present stage, a promising approach is to combine stem cell therapy with other therapies, such as gene therapy, so as to make up for some of the limitations of stem cell therapy, expand the scope of application of the therapy, and improve its efficacy. Meanwhile, we should not neglect further research on the pathogenesis. Since the specific reasons for many limitations of stem cell therapy have not yet been studied, major issues such as individual differences in PD and the neural network mechanism of PD have yet to be elucidated in depth. In conclusion, stem cell therapy for PD still has much room for development and therapeutic potential to be developed and applied by researchers.

## Data Availability

No new data were created or analyzed in this study.

## Abbreviations

The following abbreviations are used in this manuscript:

6-OHDA	6-hydroxydopamine
AD	Alzheimer’s disease
α-syn	α-synuclein
BBB	blood–brain barrier
BDNF	brain-derived neurotrophic factor
C3	complement component 3
CDNF	conserved dopamine neurotrophic factor
CJD	Creutzfeldt–Jakob disease
CNS	central nervous system
DA	dopamine
ER-phagy	endoplasmic reticulum autophagy
FAM134B	family with sequence similarity 134, member B
fNSC	fetal neural stem cell
fVM	fetal ventral mesencephalic
GBA	glucocerebrosidase
GDNF	glial cell line-derived neurotrophic factor
GLP1R	glucagon-like peptide-1 receptor
GMP	Good Manufacturing Practice
hESC	human embryonic stem cell
HGF	hepatocyte growth factor
hiPSC-DPs	human induced pluripotent stem cell-derived dopaminergic progenitors
HLA	human leukocyte antigen
hPSC	human pluripotent stem cell
hVM	human ventral mesencephalic
iNs	iPS-derived neurons
iPSC	induced pluripotent stem cell
LBs	Lewy bodies
LRRK2	leucine-rich repeat kinase 2
mDA	mesencephalic dopaminergic
MDS-UPDRS	Movement Disorder Society-sponsored revision of the Unified Parkinson‘s Disease Rating Scale
MPP	1-methyl-4-phenylpyridinium
MPTP	1-methyl-4-phenyl-1,2,3,6-tetrahydropyridine
MSC	mesenchymal stem cell
mtDNA	mitochondrial DNA
NMSS	Non-Motor Symptoms Scale
Nrf2-Keap1	nuclear factor erythroid 2-related factor 2-Kelch-like ECH-associated protein 1
nTD	non-tremor-dominant
NTN	neurturin
OFF	off-medication state
ON	on-medication state
PD	Parkinson’s disease
PDQ-39	Parkinson’s Disease Questionnaire-39
PET	positron emission tomography
PFFs	preformed fibrils
PI3K/AKT/GSK3β	phosphoinositide 3-kinase/protein kinase B/glycogen synthase kinase 3 beta
PIGD	postural instability and gait difficulty
PINK1	PTEN-induced kinase 1
POLG	DNA polymerase gamma
PRKN	parkin RBR E3 ubiquitin protein ligase
PSPN	persephin
ROS	reactive oxygen species
siRNA	mall interfering RNA
shRNA	short hairpin RNA
SN	substantia nigra
SNc	substantia nigra pars compacta
SNCA	synaptic nucleoprotein-alpha
SVF	stromal vascular fraction
TD	tremor-dominant
TnT	tunneling nanotubes
UPDRS	Unified Parkinson’s Disease Rating Scale
VEGF	vascular endothelial growth factor
VM	ventral midbrain
VPS35	vacuolar protein sorting-associated protein 35
WHO	World Health Organization
